# Territoriality Modulates the Effect of Conspecific Encounters on the Foraging Behaviours of a Mammalian Predator

**DOI:** 10.1002/ece3.71058

**Published:** 2025-03-06

**Authors:** Jeanne Clermont, Frédéric Dulude‐de Broin, Marie‐Pier Poulin, Dominique Berteaux

**Affiliations:** ^1^ Université de Sherbrooke Sherbrooke Québec Canada; ^2^ Canada Research Chair on Northern Biodiversity Université du Québec à Rimouski Rimouski Québec Canada; ^3^ Center for Northern Studies and Quebec Center for Biodiversity Science Québec Canada; ^4^ Université Laval Québec Québec Canada; ^5^ Department of Biology University of Washington Seattle Washington USA

**Keywords:** foraging, home range, intraspecific interactions, movement, territoriality, *Vulpes lagopus*

## Abstract

The probability of encountering conspecifics shapes animal behaviour, particularly for territorial individuals which often increase vigilance and scent marking when approaching home range boundaries. However, whether the foraging behaviours of territorial predators also vary with the probability of encountering neighbouring territory owners is poorly understood. We monitored 23 Arctic foxes occupying neighbouring home ranges during 2 years of contrasting resource availability on Bylot Island, Nunavut, Canada. First, based on simultaneous GPS tracking of individuals, we established which individuals used a territory by estimating the spatial distribution of the probability of encountering a neighbour within their home range. Second, using GPS and accelerometry data, we evaluated if the probability of encountering a neighbour influenced foraging behaviours, and whether this relationship differed between territorial and non‐territorial individuals. When resources were abundant, only breeding individuals excluded other foxes from a part of their home range and were thus territorial. When resources were rare, none of the foxes reproduced, and all but one were territorial. Non‐territorial individuals were less likely to cache prey in areas with a high probability of encounter, possibly to reduce cache pilfering. Territorial individuals were slightly more likely to cache prey as the probability of encountering neighbours increased, suggesting that they do not actively avoid interactions while foraging. We suggest Arctic foxes use different tactics to secure resources based on their degree of territoriality. The presence of non‐territorial predators, whose home ranges overlap those of territorial neighbours, may influence the distribution of predation risk by creating zones where predator density is high, potentially influencing predator–prey interactions.

## Graphical abstract

We monitored the movements and behaviours of Arctic foxes using neighbouring home ranges and evaluated if conspecific interactions influenced where they foraged. The probability of encountering conspecifics influenced non‐territorial Arctic foxes, which favoured caching prey where encounters were less likely to occur. Territorial individuals were less affected, suggesting territoriality is enough to secure the necessary resources for survival.

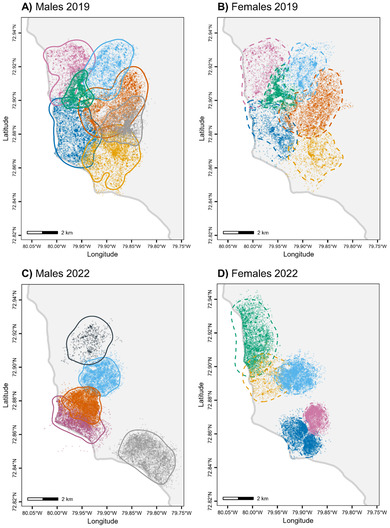

## 1 Introduction

The ability of animals to manage encounters with other individuals is key to all social and interspecific interactions, thus directly influencing reproduction, survival and ultimately fitness (Silk et al. 2003; Cresswell 2008; Wey et al. 2008). Consequently, many animal behaviours have evolved to modulate the probability of encountering other individuals. One well‐known example is how predators and prey modify their habitat use to either find or avoid each other (Sih 1984; Smith, Donadio, Pauli, Sheriff, Bidder, et al. 2019). Similarly, individuals may avoid conspecifics to decrease resource competition and become territorial, given the benefits of territoriality outweigh the costs (Maher and Lott 2000; Ord 2021). A territory represents the area of a home range that is successfully defended by one or several individuals, resulting in the territory owners having exclusive access to the defended area (Brown and Orians 1970; Powell 2000; Hinsch and Komdeur 2017). Territorial individuals will often signal space ownership through visual, auditory or olfactory signals and aggress intruders when encountered (Sillero‐Zubiri and Macdonald 1998; Giuggioli et al. 2011; Tórrez‐Herrera et al. 2020).

The intensity of territoriality is often assessed by measuring the amount of overlapping area between neighbouring home ranges (Persson et al. 2010) or the frequency with which individuals adopt territorial behaviours such as scent marking (Fawcett et al. 2013). Furthermore, the degree to which individuals of a given species are territorial can vary among and within populations (Macdonald et al. 1999; McLoughlin et al. 2000; Eide et al. 2004). Ecological variables such as the abundance, predictability and distribution of food resources influence the costs and benefits of territoriality and therefore partly explain the degree of territoriality (Maher and Lott 2000; Sells and Mitchell 2020). Notably, a cost–benefit model developed by Maher and Lott (2000) suggests that individuals should be territorial when food abundance and predictability are intermediate but non‐territorial when food abundance and predictability are below or above some thresholds (also supported by McLoughlin et al. 2000). Costs and benefits of territoriality may also vary according to predator and competitor densities (Maher and Lott 2000; Webber et al. 2023), individual characteristics such as sex (Rosell and Thomsen 2006; Fawcett et al. 2013) or other variables such as the degree of relatedness among neighbours (Persson et al. 2010; Humphries et al. 2021).

The probability of encountering neighbours varies spatially in the home range of territorial animals, with the highest probability occurring near territory boundaries. Where the probability of encountering a competitor is high, individuals should modify their behaviour to avoid potentially costly physical encounters while still signalling territory ownership more intensively (Schlägel et al. 2017). For example, when located near boundaries, white‐faced capuchins ( *Cebus capucinus* ), which defend group territories, tend to socialise less with each other (Tórrez‐Herrera et al. 2020) and travel at lower speed (Noonan et al. 2021), suggesting more vigilance, while Ethiopian ( *Canis simensis* ) and grey wolves ( *Canis lupus* ) increase scent marking (Sillero‐Zubiri and Macdonald 1998; Zub et al. 2003). An increase in time spent vigilant or in signalling territory ownership when the probability of encountering a competitor is high could further result in less time spent foraging (Laundré et al. 2001). Furthermore, the probability of encounter may directly affect specific foraging behaviour. For example, in food caching animals that are territorial, such as many canids, felids and mustelids, it may be beneficial to cache food away from competitors to reduce cache pilfering (van der Veen et al. 2020). Better understanding how intraspecific interactions and territoriality shape foraging behaviours could be particularly important for predators, as they generate predation risk landscapes that influence the dynamics of prey populations and community structure (Lima 2002; Gaynor et al. 2019; Clermont, Grenier‐Potvin, et al. 2021). For example, in systems where the predator is highly territorial and avoids encountering conspecifics, buffer zones between territories may serve as refuges for prey (Mech 1977; Lewis and Murray 1993). Prey may thus benefit from fewer predator encounters in places where territorial interference is high and could be further relieved if predators also spend less time foraging in these areas.

Modern tracking techniques help to study predator behaviours as they can locate animals at high frequencies while producing detailed behavioural classification (Nathan et al. 2012; Wilmers et al. 2015), allowing us to gain information on behaviours such as scent marking (Bidder et al. 2020), killing of prey (Studd et al. 2021) and food caching (Clermont, Woodward‐Gagné, et al. 2021). Estimating where encounters between individuals occur is, however, challenging, limiting our ability to assess how probabilities of encounter affect behaviours (Noonan et al. 2021). So far, most research has compared how animals behave in and out of overlapping areas or with respect to the distance to territory borders (assuming these variables are good surrogates for encounter probability), instead of directly estimating the spatial distribution of encounter probabilities (Rosell and Thomsen 2006; Tórrez‐Herrera et al. 2020). To address this methodological gap, Noonan et al. (2021) proposed a statistical framework using tracking data and home range estimation to evaluate the spatial distribution of the probability of encounter, termed the conditional distribution of encounters (CDE). The CDE quantifies where in space encounters are likely to occur within an individual's home range. Combining behavioural classifications with the CDE should allow us to better understand how interactions with neighbours affect the behaviours of territorial animals, further improving our understanding of important ecological processes.

We used GPS tracking and accelerometry to monitor the movements and behaviours of Arctic foxes living in a large Greater snow goose (*Anser caerulescens atlanticus*) colony on Bylot Island (Nunavut, Canada) (Clermont, Grenier‐Potvin, et al. 2021; Clermont, Woodward‐Gagné, et al. 2021) during 2 years of contrasting resource availability. At this site, foxes are socially monogamous, offer biparental care and most home ranges are occupied by a mated pair (Cameron et al. 2011). Food caching is an important dimension of the foraging ecology of Arctic foxes, which cache the majority of the goose eggs they collect (Careau et al. 2007). We first quantified the degree of summer territoriality of each studied individual by mapping the probability of encountering conspecifics other than the mate within the home range and tested the hypothesis that fox territoriality depends on the availability of food resources (H1). Following Maher and Lott's (2000) model predictions, we predicted that fewer foxes would be territorial when resources are scarce (H1: P1). We then tested the hypotheses that the probability of encountering a neighbour influences the spatial distribution of foraging behaviours within home ranges (H2), and that territoriality modulates this relationship (H3). As Arctic foxes may attack other foxes foraging within their territory and perform cache pilfering (Samelius and Alisauskas 2000; Careau et al. 2007), we predicted (H2: P1) that foxes should be less likely to hunt and cache prey where the probability of encountering a neighbour and the risk of cache pilfering are high. Lastly, we predicted (H3: P1) that the probability of encountering a neighbour should more strongly affect the foraging and caching behaviour of non‐territorial than territorial foxes, since the latter individuals might focus on excluding other foxes from their territory rather than avoiding encounters.

## 2 Materials and Methods

### 2.1 Study System

We worked in 2019 and 2022 in the southwest plain of Bylot Island (72°53′ N, 79°54′ W) in Sirmilik National Park of Canada (Nunavut), where the Arctic fox is the main terrestrial predator. Arctic foxes at this site are highly range resident (i.e., they use a stable home range), they usually share a home range with their mating partner and most individuals show low home range overlap with their neighbours (Grenier‐Potvin et al. 2021). They bark and scent mark to indicate territory ownership, and they chase intruders (Eberhardt et al. 1982). In addition to selecting habitats suitable to their main prey, they avoid home range borders, potentially to minimise interactions with their neighbours (Grenier‐Potvin et al. 2021).

On Bylot, Arctic foxes rely mostly on small prey, such as lemmings ( *Lemmus trimucronatus* and *Dicrostonyx groenlandicus* ). They also feed on greater snow goose eggs when their home range overlaps Bylot's large colony of more than 20,000 nesting pairs (Bêty et al. 2001). In 2022, however, nesting goose density was unusually low throughout the whole colony (density estimated from random plots: 128 nests/km^2^ in 2022, 438 nests/km^2^ in 2019 and an average of 248 nests/km^2^ between 2000 and 2019; Cadieux et al. 2023). Foxes also opportunistically prey on the nests of other ground‐nesting birds (Duchesne et al. 2021). Lemming density fluctuates cyclically (Gruyer et al. 2008) and was moderate in 2019 and very low in 2022, as determined by capture–recapture methods (Fauteux et al. 2015; Duchesne et al. 2021; Cadieux et al. 2023). The snow goose incubation period lasts 23 days from mid‐June to early July, during which foxes collect eggs for later consumption (Samelius et al. 2007). In years of low to moderate lemming densities, goose eggs represent the majority of prey collected by foxes during the goose incubation period (Careau et al. 2007). Foxes cache up to 90% of the goose eggs they collect, and caches are located 85 m (median) from the nest (Careau et al. 2007). They also recache ca. 60% of goose eggs, and the recovery rate (for recaching or consumption) is highest at the end and after the goose incubation period (Careau et al. 2008).

Furthermore, access to the snow goose colony influences fox probability of reproduction (Chevallier et al. 2020). Despite interindividual variations, the phenology of fox reproduction largely coincides with that of geese, with cubs remaining in the den during the goose egg incubation period and progressively emerging and becoming independent from the den during goose brooding (Grenier‐Potvin et al. 2021). Due to low densities of both lemmings and goose nests in 2022, none of the studied foxes reproduced that year, contrary to 2019 when many foxes reproduced.

### 2.2 Arctic Fox Tracking

In May and June, we captured 13 foxes in 2019 and 10 foxes in 2022 in the snow goose colony using Softcatch #1 padded leghold traps (Oneida Victor Inc. Ltd., Cleveland, OH, USA). We determined the individuals' sex from genital characteristics and fitted them with four coloured ear tags and a GPS accelerometer collar (95 g, ca. 4% of body mass; Radio Tag‐14, Milsar, Romania) for subsequent identification and tracking. Arctic fox capture techniques and immobilisation procedures were approved by the UQAR Animal Care Committee (CPA‐64‐16‐169 R3) and field research was approved by the Joint Park Management Committee of Sirmilik National Park of Canada (SIR‐2018‐28021).

**FIGURE 1:**
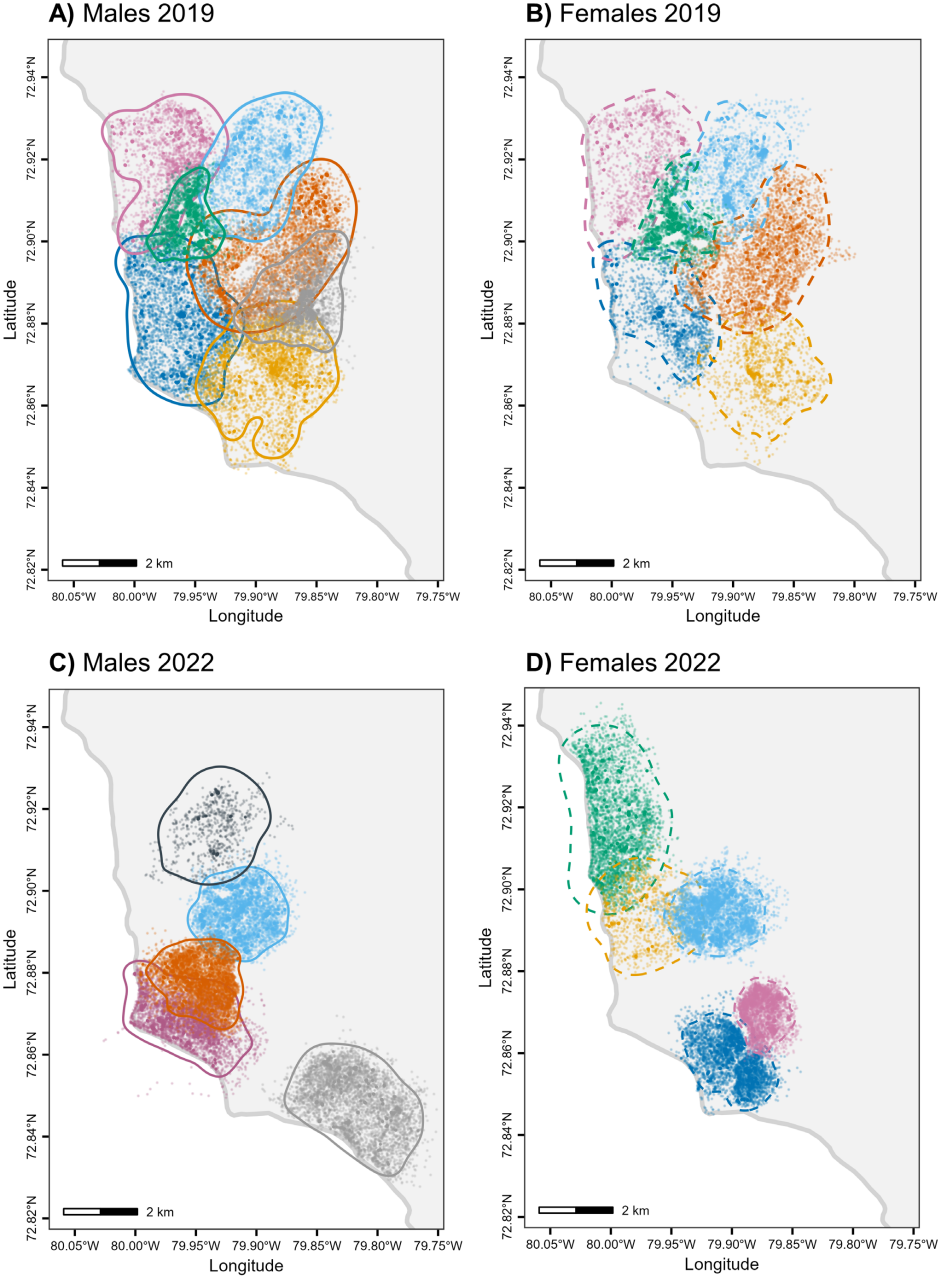
GPS locations (dots) of male and female Arctic foxes tracked from 11 June to 4 July 2019 when resources were abundant (*n* = 67,109 locations from 13 individuals, panels A and B) and from 19 June to 12 July 2022 when resources were scarce (*n* = 47,631 locations from 10 individuals, panels C and D) at a 4‐min fix interval on Bylot Island, Nunavut, Canada. Solid (males) and dashed (females) lines are 95% home range contours obtained through autocorrelated kernel density estimation. For each year, the same colours are used for members of a given fox pair. Home ranges are located near the coast of Bylot Island, shown by the thick grey line.

**FIGURE 2:**
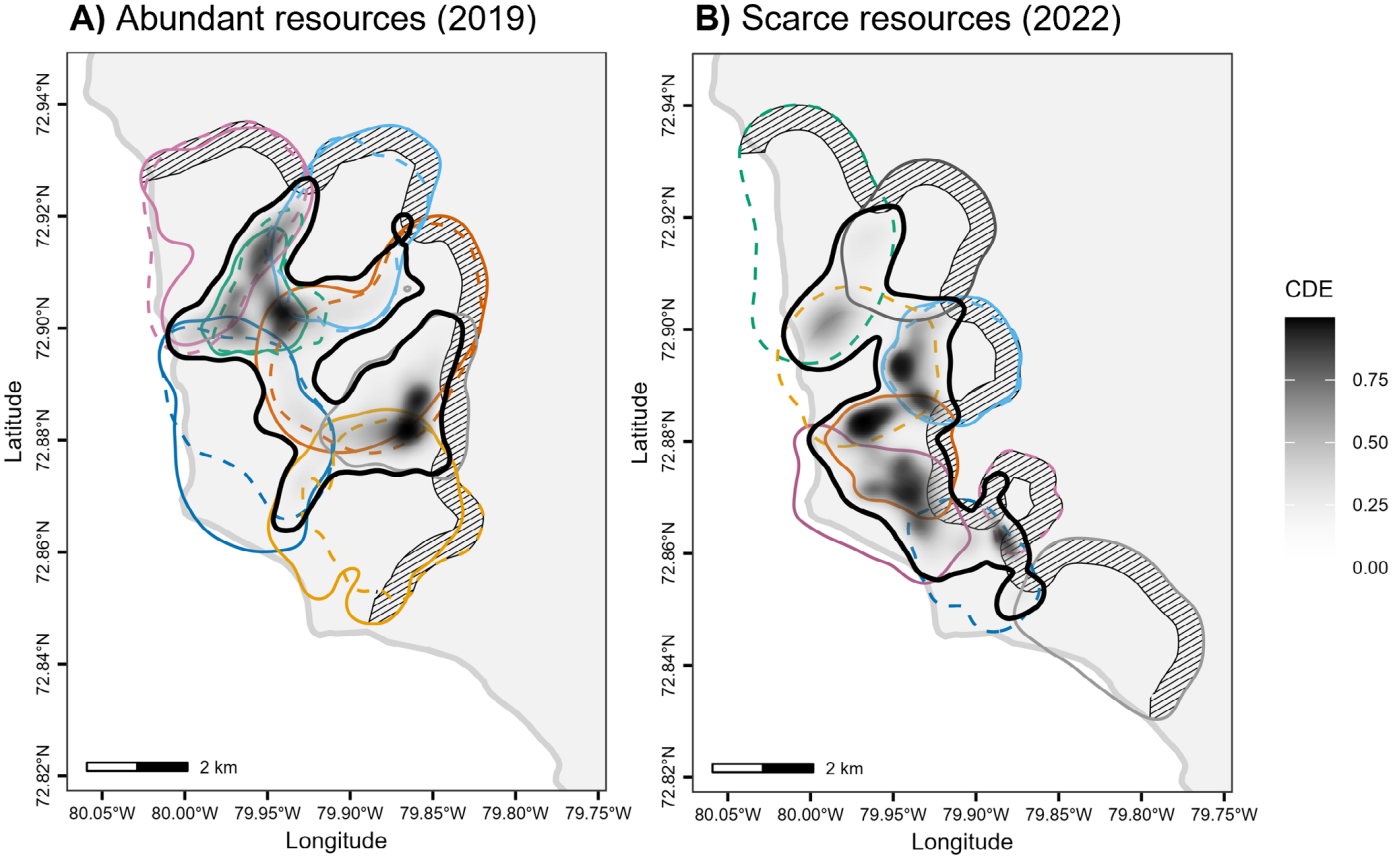
Conditional distribution of encounters (CDE) among (A) 13 Arctic fox neighbours in 2019 when resources were abundant and (B) 10 Arctic fox neighbours in 2022 when resources were scarce, tracked with GPS, excluding interactions between pair members. The greyscale gradient indicates CDE values, where higher (darker) values suggest higher probabilities of encounter. The thick black line is the 95% CDE contour and coloured lines are 95% home range contours. Striped areas represent the 500 m inner buffer of study area boundaries where interactions with non‐monitored foxes could have occurred. Males and females are represented by solid and dashed lines, respectively, and members of a given pair are identified by the same colour within each year.

In 2019, the 13 tracked foxes consisted of six neighbouring pairs plus one individual, and the area encompassing all their home ranges formed our 2019 study area (Figure 1A,B). In 2022, the 10 tracked foxes included one pair for which both individuals were tracked, and eight pairs for which only one individual of the pair was tracked, and the area encompassing all their home ranges formed our 2022 study area (Figure 1C,D; also see Figure 2B where the 2022 study area is more easily identifiable). Only one individual was monitored during both years. We determined the reproductive status of individuals based on whether automated cameras recorded cubs at individuals' den (Cameron et al. 2011). Daily observations and automated cameras at fox dens confirmed that all foxes within our study area were tracked in 2019 and at least one individual per pair in 2022. We collected one GPS location every 4 min and one 30‐s burst of accelerometry (50 Hz) every 4.5 min in 2019 and every 4 min in 2022 (Clermont, Woodward‐Gagné, et al. 2021). We divided the 30‐s bursts of accelerometry into 10 3‐s sequences and, using behavioural observations and a random forest algorithm, we assigned to each sequence one of four behaviours: running, walking, digging and motionless (see analytical details in Clermont, Woodward‐Gagné, et al. 2021). During the goose incubation period, digging behaviour of foxes is mainly associated with goose egg caching, but may also occasionally reflect caching and handling of other prey species as well as cache recoveries (Clermont, Woodward‐Gagné, et al. 2021). As most captured prey are cached (Careau et al. 2007), fox digging behaviour is more generally a proxy of foraging activity.

### 2.3 Data Analysis

#### 2.3.1 Estimation of Home Ranges

To define Arctic fox summer home ranges, we used GPS data collected from 11 June to 4 July 2019, and 19 June to 12 July 2022, corresponding to goose egg incubation periods. Prior to home range estimation, we confirmed home range residency for each individual using variogram analysis (Fleming et al. 2014) and excluded location data resulting from extra‐territorial trips as it affects home range estimation (Calabrese et al. 2016). Indeed, during the study period, some individuals made a few extra‐territorial trips going up to 25 km away from the centre of the home range. These ‘outliers’ were identified by evaluating distances between points and the home range core, and a cut‐off distance was determined visually for each individual (see Appendix A for further details). We excluded 11%, 9%, 6% and 4% of datapoints for four individuals that performed extra‐territorial trips (three individuals in 2019 and one in 2022). The remaining individuals showed high range residency, and we excluded < 1% of their GPS locations.

We then fitted range‐resident continuous‐time movement models to the data of each individual (model selection resulted in the Ornstein–Uhlenbeck Foraging [OUF] process used for each individual) to control for autocorrelation in both speed and location (Fleming et al. 2014; Calabrese et al. 2016). Then, we estimated home range areas using autocorrelated kernel density estimation (AKDE; Fleming et al. 2015). AKDE is more accurate than other home range estimators for autocorrelated location data (Noonan et al. 2019). The complete workflow we used is detailed in Silva et al. (2021) and was performed using the package ctmm (v0.6.2; Calabrese et al. 2016) in R 4.1.0 (R Development Team 2021). We used a linear mixed model (lme4 package, v1.1‐35.1; Bates et al. 2015) to assess the effects of sex and year (both as fixed factors) on home range area. To account for non‐independence within pairs, we included pair ID as a random effect.

#### 2.3.2 Estimation of Probability of Encounter Among Neighbours

Following Noonan et al. (2021), we estimated the conditional distribution of encounters (CDE) for each year separately (package ctmm v0.6.2). The CDE estimates the spatial distribution of encounter events in the environment as the normalised product of AKDE home range estimates. The probability of encounter estimated by the CDE thus represents the probability that more than one individual uses a specific area, but not necessarily at the same time. A CDE value of 1 at a given location indicates there is a 100% chance that more than one individual use that location, and thus that the probability of encounter is high, and a value of 0 indicates that only one individual uses that location, meaning individuals should never encounter each other at that location. Since we were interested in encounters among neighbours, we excluded interactions between mates within their shared home range. We then determined the degree of territoriality of each fox and compared the proportion of territorial individuals between years (H1: P1). A fox was considered territorial if at least some part of its home range was outside of the 95% CDE (Noonan et al. 2021), excluding a buffer within the boundaries of study areas. Indeed, some interactions inevitably occurred at home range boundaries located at the edge of the 2019 and 2022 study areas (but not near the coastline, Figures 1 and 2), where neighbouring foxes were not tracked, leading to a local underestimation of CDE values. We considered two buffer sizes, a less conservative one of 500 m and a more conservative one of 1 km. We present results based on the 500 m buffer in the main text. Using a 1‐km buffer instead of a 500‐m buffer would have led to the same classification (territorial yes/no) of all individuals but one in 2022 (female BJOV; see Appendix B).

#### 2.3.3 Arctic Fox Prey Caching Behaviour

We used our accelerometry behavioural classification to determine whether individuals engaged in digging (indicating prey caching, cache recovery events and more generally foraging activity) during each 30‐s accelerometry burst. At least one out of the 10 3‐s accelerometry sequences had to be assigned to the digging behaviour to consider that the fox engaged in digging in that burst. Each 30‐s burst was then associated with the closest GPS location, provided the time stamp of the GPS location was within 30 s of the start or end of the burst. Bursts occurring at less than 50 m of a den were excluded as digging may then reflect den maintenance rather than prey caching.

We excluded from analyses all accelerometry bursts occurring within 500 m of the boundaries of study areas since interactions with unknown foxes may then occur (Appendix B). To verify whether buffer size influences results, we (1) repeated the analyses described in the next section by excluding the individual of 2022 that would have been considered non‐territorial when using a 1‐km buffer (female BJOV), and (2) repeated our analyses by excluding locations falling within a 1‐km buffer instead of a 500‐m one (Appendix B). In both cases, we obtained similar results.

#### 2.3.4 Effect of the Probability of Encountering a Neighbour on Fox Behaviours

To test the influence of the likelihood of encountering a neighbour on the probability of engaging in foraging behaviours, we used generalised additive mixed models (GAMM) with a binomial error distribution and logit link function (‘gam’ function of the R package mgcv, v1.9‐0; Wood 2017) and modelled the probability of engaging in digging (0 = no digging, 1 = digging; H2: P1) with respect to the probability of encounter. As not all foxes experienced the same range of probability of encounter, we centred the probability of encounter values to a mean of 0 within each fox home range. We included in the model an interaction between the categorical variable of territoriality (yes/no) and the probability of encounter to test whether territoriality modulated the effect of the probability of encounter on the probability of digging (H3: P1). We included individual sex as another fixed effect. As random intercepts, we included fox identity, year and ordinal date nested in year to account for repeated measurements on the same dates and potential unmeasured shared conditions. In contrast to the home range analysis, we did not include pair ID as a random intercept for the foraging analysis as foxes generally hunt alone and their foraging behaviour could be independent from that of their mate. To further address temporal autocorrelation in our data set and control for nonlinear temporal trends in the probability of digging, we also added a cyclic cubic spline function of the numeric time of the day and a thin plate regression spline function of the ordinal date, with 10 basis dimensions for each function (Pedersen et al. 2019). Continuous covariates were centred and standardised to facilitate the interpretation of model estimates (Schielzeth 2010). We verified model assumptions and the independence of residuals using the R package DHARMa (v0.4.3; Hartig 2021).

In addition to obtaining a yearly, colony‐level estimate of nesting goose density (as described in Section 2.1 above), we also performed detailed field surveys to estimate nesting goose density (individual geese/ha) within each fox home range in 2019, by mapping the contours of relatively homogeneous nesting goose patches (Grenier‐Potvin et al. 2021). We did not perform these surveys in 2022 as nest density was very low throughout the study area; therefore, we could not include fine‐scale nesting goose density as a covariate in the model. As nesting goose density was previously shown to affect fox probability to dig (Clermont, Woodward‐Gagné, et al. 2021), we verified whether including this covariate in the model affected results using data from 2019 exclusively. We obtained similar results with and without this covariate (Appendix C).

Results are expressed using evidence‐based language (Muff et al. 2021) where *p*‐value ≥ 0.1 suggests no evidence, *p*‐value = ]0.05, 0.1] suggests weak evidence, *p*‐value = ]0.01, 0.05] suggests moderate evidence, *p*‐value = ]0.01, 0.001] suggests strong evidence, and *p*‐value < 0.001 suggests very strong evidence.

## 3 Results

### 3.1 Conditional Distribution of Encounters in Fox Home Ranges

Home range areas averaged 7.93 ± 3.15 (SD) km^2^ (Figure 1, *n* = 23). We found no evidence that home range area differed between sexes (Table 1, average male home range area = 8.27 ± 3.12 (SD) km^2^, *n* = 12, average female home range area = 7.56 ± 3.28 (SD) km^2^, *n* = 11) and years (Table 1, average home range area in 2019 = 9.03 ± 3.12 (SD) km^2^, *n* = 13, average home range area in 2022 = 6.51 ± 2.70 (SD) km^2^, *n* = 10).

**TABLE 1:** Estimates of a linear mixed model predicting Arctic fox home range area (*n* = 23), as a function of sex and year of observation, with pair identity as a random effect.

Term	Estimate	SE	*t*	*p*
(Intercept)	8.29	1.15	7.23	< 0.001
Sex [male]	1.04	0.57	1.83	0.109
Year [2022]	−2.17	1.48	−1.46	0.167

**FIGURE 3:**
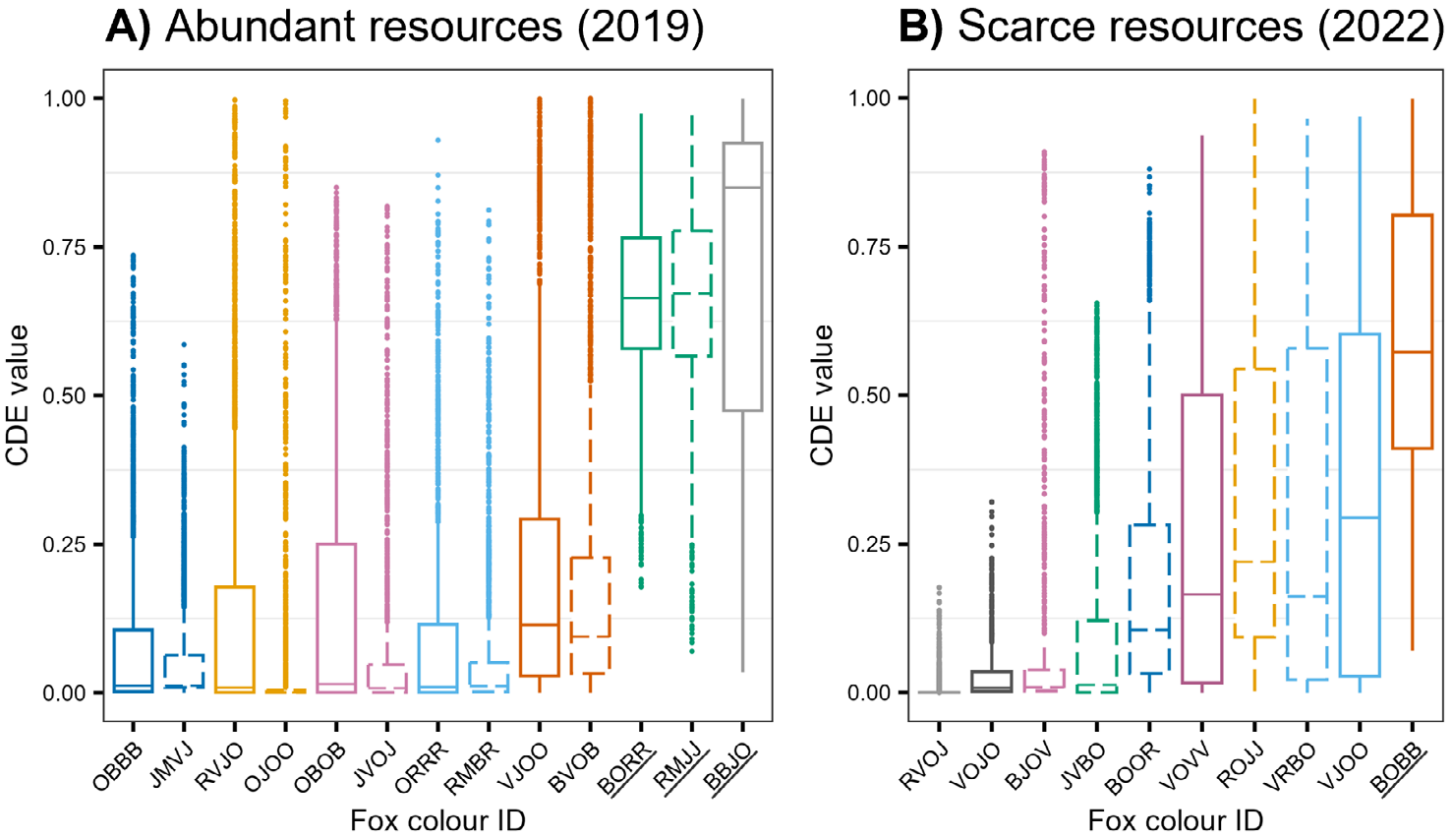
CDE values (probability of encountering a neighbour) observed at each GPS location from Figure 1, for each fox in (A) 2019 and (B) 2022. Fox colour IDs reflect the unique combinations of colours of the four ear tags (e.g., OBBB: Orange‐blue‐blue‐blue). Boxplot colours used for each individual are the same as in Figures 1 and 2. Males and females are represented by solid and dashed lines, respectively, and each year's members of a given pair are identified by the same colour. IDs of individuals categorised as non‐territorial are underlined. Boxplots show first quartile, median, and third quartile. Lower and upper whiskers extend, respectively, to the lowest and highest values within the interquartile range multiplied by 1.5. Points represent values outside this range.

When resources were abundant (2019), three individuals (green female RMJJ, green male BORR and grey male BBJO) were non‐territorial as they had all of their home range located within the 95% CDE contour if we ignore 500 m study area edges (Figures 2A and 3A). These three foxes did not reproduce in 2019. In contrast, all other 10 individuals were territorial, having a considerable area of their home range outside the 95% CDE contour, where the probability of encounter with a neighbour is null (Figures 2A and 3A). These 10 individuals reproduced in 2019. When resources were rare (2022), all individuals but one (dark orange male BOBB) were territorial (Figures 2B and 3B). None of the foxes reproduced in 2022. We thus find no support for an effect of resource abundance on territoriality (H1: P1) as 10/13 and 9/10 individuals were territorial in 2019 (high resource abundance) and 2022 (low resource abundance), respectively.

### 3.2 Effect of the Probability of Encountering a Neighbour on Fox Caching Behaviour

**FIGURE 4:**
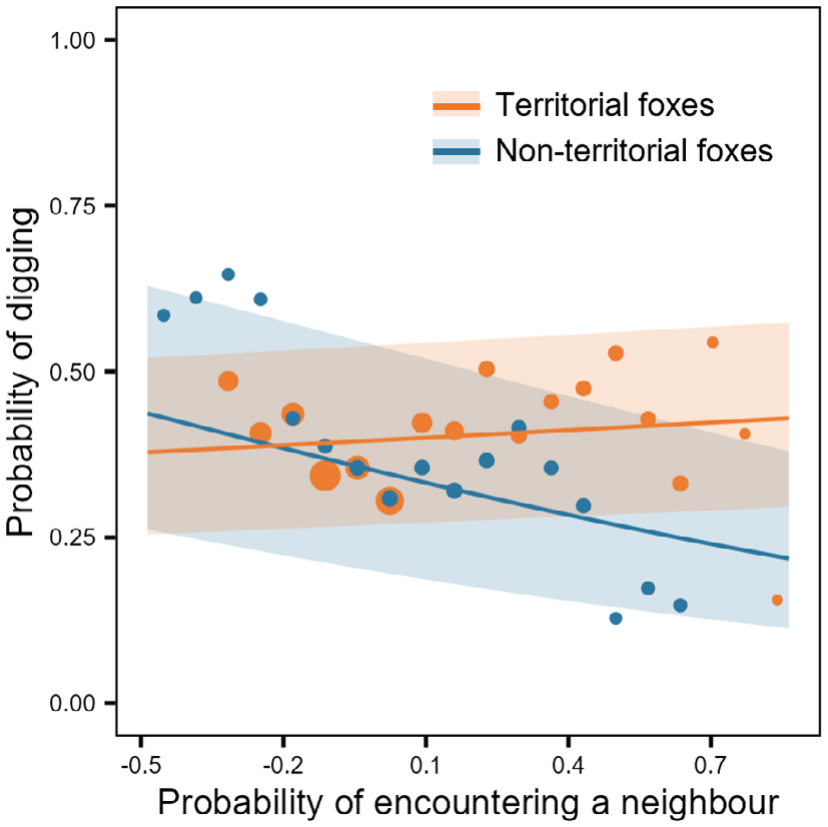
Predicted effects of the probability of encountering a neighbour on probability of fox digging (0 = no digging, 1 = digging, *n* = 63,093 accelerometry bursts). The probability of encountering a neighbour was obtained from conditional distributions of encounters (Figure 2). Dots are raw values of probability of digging averaged over equally sized intervals of probability of encountering a neighbour. Size of dots reflects number of observations in each interval. Shaded areas represent 95% confidence intervals (CI). Orange dots, lines and CI refer to territorial individuals (*n* = 19), and blue dots, lines and CI refer to non‐territorial individuals (*n* = 4).

**FIGURE A6:**
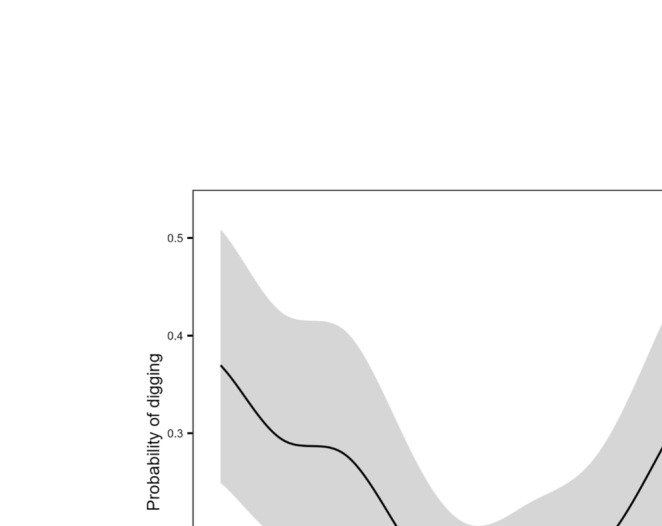
Predicted effect of the time of day on probability of fox digging (0 = no digging, 1 = digging, *n* = 63,093 accelerometry bursts). Model specifications are in Table 2.

Non‐territorial foxes avoided to engage in digging in places with high encounter probability (strong evidence; Table 2; Figure 4; supporting H2: P1). This avoidance was not reflected in the behaviour of territorial foxes, which were in contrast slightly more likely to dig at high probability of encounter (strong evidence; Table 2; Figure 4; contradicting H2: P1). However, the effect size for territorial foxes was small, and there was no evidence of an effect when modelling data from 2019 alone (Appendix C). Overall, the probability of encountering a neighbour had a larger impact on the behaviour of non‐territorial than territorial foxes (Figure 4; supporting H3: P1). We found no evidence of an effect of sex (Table 2), strong evidence of a nonlinear effect of time of day on the probability of digging (illustrated in Appendix D: Figure A6), but no effect of the smooth term ordinal date (Table 2). The random effects year, ordinal date (factor) and fox ID were all significant (Table 2).

**TABLE 2:** Estimates of a generalised additive mixed model predicting the probability of fox digging (*n* = 63,093 accelerometry bursts from 23 individuals), with a binomial error distribution and a logit‐link function. We included as parametric terms (fixed effects) the probability of encountering a neighbour, whether the fox was territorial, an interaction between the probability of encountering a neighbour and whether the fox was territorial and the individual's sex. We included splines for numeric time of day (ToD) and ordinal date (day), and the random effects year, ordinal date (day_factor) nested in year and fox identity (Fox ID).

Component	Term	Estimate	SE	*z*	*p*
1. Parametric coefficients	(Intercept)	−0.58	0.42	−1.38	0.168
	Probability of encountering a neighbour	0.04	0.01	3.68	< 0.001
	Territorial yes/no [no]	−0.22	0.33	−0.65	0.515
	Sex [male]	−0.11	0.26	−0.43	0.666
	Prob encounter: Territorial yes/no [no]	−0.22	0.02	−9.49	< 0.001
**Component**	**Term**	**edf**	**Ref.df**	**Chi.sq**	***p***
2. Smooth terms	s(ToD)	7.51	8.00	9597.33	< 0.001
	s(day)	3.31	3.51	2.72	0.436
	s(year)	0.99	1.00	34,859.99	< 0.001
	s(day_factor, year)	31.69	45.00	210.17	< 0.001
	s(Fox ID)	18.73	19.00	3683.47	< 0.001

## 4 Discussion

Using high‐throughput tracking technologies, we estimated the spatial distribution of the probability of encounter among Arctic foxes using neighbouring home ranges. We benefited from a natural situation where most, but not all, individuals were territorial, thus enabling us to evaluate the effect of territoriality on the spatial distribution of foraging behaviour. Non‐territorial individuals responded to the variation in the probability of encountering a neighbour by shifting the distribution of digging behaviour away from the places where encounters were most likely to occur. On the other hand, territorial individuals were slightly more likely to dig where the probability of encountering a neighbour was high. Our results suggest that territoriality may reduce risks associated with the probability of encountering a neighbour and could relax the need for behavioural adjustments when foraging. Despite modern technology, it remains highly challenging to simultaneously track the movements and foraging behaviours of many neighbouring vertebrate predators at high resolution, in a natural setting and over several weeks. Our sample of 23 individuals, each tracked for more than 3 weeks, is therefore of high value. Our study provides valuable insights into how intraspecific interactions may influence the spatial distribution of predator foraging.

### 4.1 The Degree of Territoriality Varies Among Foxes

Variation in the distribution, abundance and predictability of resources often explains among‐individual differences in territoriality (Maher and Lott 2000; Sells and Mitchell 2020). For example, Eide et al. (2004) observed that, in Arctic foxes of Svalbard, high home range overlap occurred near the coast where seabird colonies are concentrated. On the other hand, little home range overlap occurred inland where reindeer ( *Rangifer tarandus* ) carcasses are scattered and unpredictable. In our study, we do not suspect that variation in prey distribution, abundance or predictability explains among‐individual differences in territoriality within years, as all studied foxes lived near the centre of the goose colony where nest abundance is usually high and habitats favourable to lemmings. However, we used two contrasted years of data, with 1 year of moderate lemming abundance and typical goose density for this study area (2019), and 1 year of low lemming abundance and low goose density (2022). In 2019, although resource abundance was high, not all foxes reproduced and used a territory. This suggests that using a territory may be necessary to secure enough resources to ensure the survival of young, even when food is abundant (Webb et al. 2012; López‐Bao et al. 2014). On the other hand, for non‐breeding individuals, the costs of defending a territory may have been too high compared to the benefits when resources are abundant, as predicted by Maher and Lott's (2000) cost–benefit model of territoriality.

Although resources were scarce in 2022, all foxes but one could exclude neighbours from part of their home range, which seems to contradict Maher and Lott's (2000) model that predicts no territoriality when resources are very rare. Similarly, Warret Rodrigues and Roth (2023) observed low home range overlap among neighbouring Arctic and red foxes in a resource‐poor environment. Our results suggest that, in 2022, food abundance in the colony was still high enough for territoriality to remain beneficial. In fact, foxes in the colony might rely on stored eggs from the previous year (Samelius et al. 2007), which could attenuate the effects of low resources. However, although a high abundance of resources may permit non‐breeding individuals to forego being territorial, defending a territory may remain beneficial under low or very low resource abundance, especially when the abundance of resources varies cyclically through time. Similarly, López‐Bao et al. (2014) observed that home range overlap among Iberian lynx ( *Lynx pardinus* ) remains low across a gradient of prey availability. This obstinate strategy (sensu von Schantz 1984), that is, maintaining a territory even when resources are rare, could be beneficial if it allowed to secure a home range in areas where resources are usually high, such as the snow goose colony on Bylot Island. Therefore, it may remain beneficial for foxes to defend a territory in the goose colony even during years of low goose nesting density, to maintain territory ownership and maximise chances of future reproduction (López‐Bao et al. 2014).

### 4.2 Variable Effect of the Probability of Encountering a Neighbour on Arctic Fox Foraging Behaviours

Foraging and caching prey in places where the probability of encountering a neighbour is high can increase the risk of injury or cache pilfering (Samelius and Alisauskas 2000; van der Veen et al. 2020). Therefore, Arctic foxes should be more likely to forage and cache prey where they are less likely to encounter neighbours. In fact, a previous habitat selection analysis showed that when they are in an active state, Arctic foxes avoid using the edges of their home range (Grenier‐Potvin et al. 2021), suggesting spatial avoidance of areas with a high probability of encountering neighbours. However, it was unknown whether foxes also adjust their foraging behaviour when located within these areas. We found that the level of territoriality influenced how Arctic foxes responded to variation in the probability of encountering a neighbour. Non‐territorial foxes had a higher probability to dig where the probability of encountering neighbours was low. For these individuals without exclusive home range, foraging and caching prey where the risk of cache pilfering is low may increase foraging efficiency. Although during goose incubation period, digging events should mostly reflect prey caching, we cannot differentiate caching events from prey captures, cache recoveries and recaching events (Clermont, Woodward‐Gagné, et al. 2021). Therefore, our results may also indicate that non‐territorial individuals could favour recaching away from their neighbours, in safer sites. Caching and recaching prey in ‘out of view’ sites are in fact common cache protection strategies in other species such as corvids (Dally et al. 2006). Careau et al. (2007) also showed that Arctic fox recaches goose eggs away from where they were collected, potentially to secure eggs in sites where chances of pilfering are lower (e.g., closer to the den).

Territorial foxes, on the other hand, were more likely to dig as the probability of encountering a neighbour increased, but this effect was small and no longer significant when modelling data from 2019 alone. By defending a territory, territorial individuals had a null encounter probability in substantial parts of their home range. This likely reduced the need to modulate caching behaviours, a potential benefit of establishing a territory. Still, territorial individuals could have withheld from foraging and caching in the few parts of their home range where encounter probability was not null. However, our results suggest that they did not, and rather that they slightly increased their use of digging behaviour, potentially because maintaining (and therefore using) their territory is more important than avoiding the risk of injury or cache pilfering by conspecifics. Greater probability of digging where the probability of encounter is high could also indicate a higher rate of recaching in these areas. Territorial individuals are likely more dominant and better suited to win an encounter than non‐territorial individuals (Stamps and Krishnan 2001). It might therefore be beneficial for these individuals to actively use all parts of their home range at the risk of injury and cache pilfering rather than leaving some areas undefended. Our results suggest that territoriality could be enough to secure the necessary resources for survival and reproduction.

### 4.3 Limits of the Study

Animals most likely exhibit a continuous range of territorial behaviours. Therefore, there is likely some variation in the degree of territoriality among individuals that could not be captured by our binary classification. Furthermore, in addition to spatial adjustments of behaviours, Arctic foxes may modify their foraging behaviours temporally to avoid encounters, which the CDE did not allow to evaluate (Noonan et al. 2021). Similarly, prey species access resources in high‐predation risk areas at times of the day when predators are less active (Kohl et al. 2018; Smith, Donadio, Pauli, Sheriff, and Middleton 2019). Future studies should include temporal partitioning when estimating the probability of encounter to help uncover the different tactics territorial animals use to avoid intraspecific interactions while foraging.

### 4.4 Implications for Predator–Prey Interactions

Arctic foxes have important top‐down effects in the tundra and generate multiple predator‐mediated interactions among prey (Bêty et al. 2002; Legagneux et al. 2012; Duchesne et al. 2021). For example, nest survival of American golden plover ( *Pluvialis dominica* ) is lower within the snow goose colony, where foxes use smaller home ranges and are thus present at higher densities (Dulude‐de Broin et al. 2023). At fine spatial scales, Arctic fox movements generate a predation risk landscape influencing the behaviour and nest distribution of several migratory birds (Clermont, Grenier‐Potvin, et al. 2021). It is therefore of strong interest to identify the factors explaining Arctic fox density and where they choose to forage. In this study, we observed that non‐territorial individuals avoid caching prey where the probability of encountering a neighbour is high. As most captured prey are also cached (Careau et al. 2007), our results suggest non‐territorial individuals forage less intensively in areas likely used by other individuals. However, most individuals were territorial, and the effect of the probability of encounter on the caching behaviour of this group was opposite, but weak, suggesting intraspecific interactions within home ranges may not have a strong effect on the overall spatial distribution of predation risk. Still, the presence of non‐territorial individuals, whose home ranges overlap those of their territorial neighbours, creates zones where fox density is high, which can increase the risk of predation (Clermont, Grenier‐Potvin, et al. 2021; Dulude‐de Broin et al. 2023). As such, we hypothesise that the greatest risk of predation should be located within the home range of non‐territorial individuals.

## 5 Conclusion

We found that not all Arctic foxes on Bylot Island used a territory. We highlight that because the costs and benefits of territoriality may differ among individuals in a population, alternative behavioural tactics may emerge from non‐territorial individuals to secure resources. Including the conditional distribution of encounters into habitat selection analyses would further help to better understand how different individuals deal with both the physical and social environments when using their habitat, and how they compromise between the needs to acquire valuable resources and avoid competitors. Finally, a spatial configuration in which the home ranges of non‐territorial individual predators overlap those of their territorial neighbours may influence the distribution of predation risk, which could modulate the distribution and behaviour of prey and the structure of communities.

## Author Contributions

**Jeanne Clermont:** conceptualization (lead), data curation (equal), formal analysis (lead), investigation (equal), methodology (equal), project administration (supporting), visualization (lead), writing – original draft (lead), writing – review and editing (equal). **Frédéric Dulude‐de Broin:** conceptualization (supporting), data curation (equal), formal analysis (supporting), investigation (equal), methodology (equal), writing – review and editing (equal). **Marie‐Pier Poulin:** conceptualization (supporting), data curation (equal), formal analysis (supporting), investigation (equal), methodology (equal), visualization (supporting), writing – review and editing (equal). **Dominique Berteaux:** conceptualization (supporting), data curation (equal), funding acquisition (lead), investigation (equal), methodology (supporting), project administration (lead), writing – review and editing (equal).

## Conflicts of Interest

The authors declare no conflicts of interest.

## Supporting information


Appendix S1

## Figures and Tables

**FIGURE A1:**
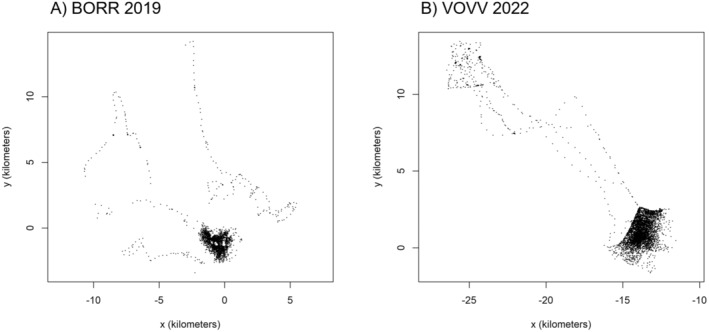
GPS locations for (A) individual BORR in 2019 and (B) individual VOVV in 2022, that performed a few extra‐territorial trips during the study period. *X* and *Y* axes show distance from home range core.

**FIGURE A2:**
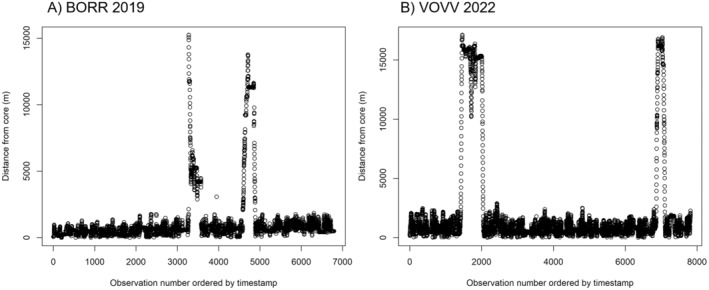
Distances between GPS locations and the core of the home range for (A) individual BORR in 2019 and (B) individual VOVV in 2022.

**FIGURE A3:**
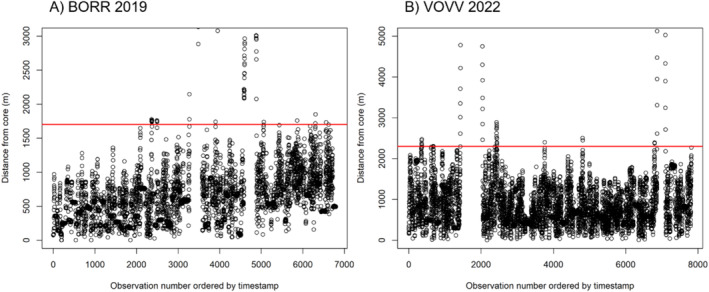
Zoom in on distances between GPS locations and the core of the home range for (A) individual BORR in 2019 and (B) individual VOVV in 2022. Red lines show the 1700 and 2300 m cut‐offs, respectively.

**FIGURE A4:**
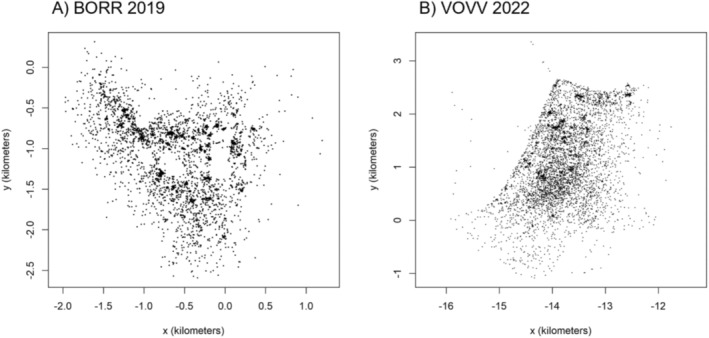
GPS locations for (A) individual BORR in 2019 and (2) VOVV in 2022 after the removal of extra‐territorial trips.

**FIGURE A5:**
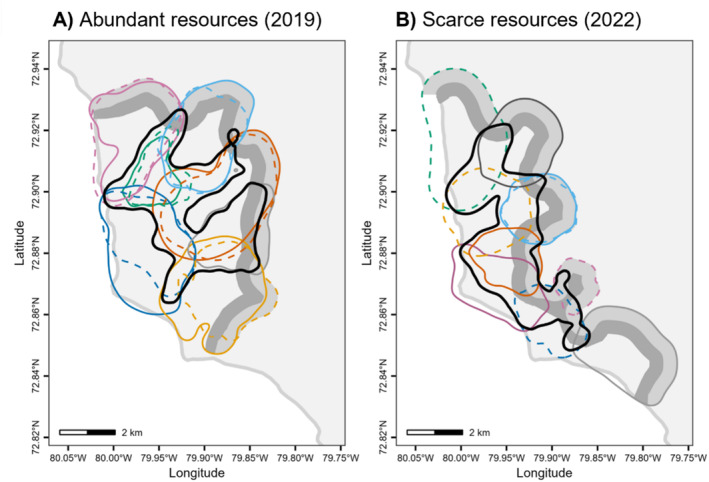
Study area with 95% autocorrelated kernel density estimation contours for (A) seven male and six female Arctic foxes in 2019 when resources were abundant and (B) five male and five female Arctic foxes in 2022 when resources were scarce. The light grey‐shaded area represents a 500‐m inner buffer of study area boundaries where observations were excluded, while the dark grey‐shaded area indicates a 1‐km buffer. The thick black line is the 95% CDE contour used to determine whether foxes were territorial, and coloured lines are 95% AKDE contours. Males and females are represented by solid and dashed lines, respectively, and members of a given pair are identified by the same colour (within year).

## Data Availability

Arctic fox GPS and accelerometry data are available through the Movebank Data Repository (Movebank Study ID 1241071371 and 2602389249). Data and scripts used for generalised additive mixed models are available as Supporting Information.

## Appendix A Workflow to Exclude GPS Locations Associated With Extra‐Territorial Trips for Home Range Estimation

Some individuals performed extra‐territorial trips (Figure A1), which affected home range estimation. We considered these GPS locations associated with extra‐territorial trips as outliers and identified them by calculating the distance between the core of the home range and each GPS location in the R package ctmm with the function ‘outlie’ (Calabrese et al. 2016). Figure A2 shows distance values for the individual BORR in 2019 (green male in Figure 1A of the main text) and VOVV in 2022 (dark purple male in Figure 1C of the main text). Zooming in on lower distance values helped to determine cut‐offs for individuals travelling far from their home range core (Figure A3). For BORR, we removed GPS locations > 1700 m from the home range core, which removed 9% of its original dataset. For VOVV, we removed GPS locations > 2300 m from the home range core, which removed 11% of its original dataset. These thresholds were determined visually so that extra‐territorial trips disappeared from the spatial representation of the individual's movements (Figure A4). We also removed 4% of GPS locations for RMJJ in 2019 (green female in Figure 1B), 6% for BBJO in 2019 (grey male in Figure 1A), and < 1% for all others.

## Appendix B Exclusion of Datapoints Near Study Area Boundaries

Interactions with foxes that were not tracked occurred on the outer margin of our study area, thus biasing negatively our CDE estimates. For that reason, when we determined whether foxes were territorial (i.e., whether they had a part of their home range located outside of the 95% CDE), we excluded areas within 500 m of study area boundaries (Figure 2). This led to three individuals considered as non‐territorial in 2019 (green female RMJJ, green male BORR and grey male BBJO, Figure 2), and one in 2022 (dark orange male BOBB, Figure 2). Using a larger buffer size of 1 km would have led to the pink female BJOV in 2022 to also be considered as non‐territorial, as the 1 km buffer overlaps all her home range (Figure A5).

To test the effect of the probability of encountering a neighbour on Arctic fox behaviour, we excluded from our dataset all locations falling within the 500 m inner buffer of the study area and considered that BJOV of 2022 was territorial (results in Table 2). Due to uncertainty in BJOV's territorial degree, we repeated the analyses while excluding this individual. Results are similar to those shown in Table 2 (Table A1).

We also verified whether our choice of buffer size influenced the results by repeating models, this time excluding locations falling within an inner buffer of 1 km instead of 500 m. This also resulted in excluding all locations of individual BJOV in 2022 as they were all within the 1 km buffer (Figure A5). Results are similar to those shown in Table 2 (Table A2).

**TABLE A1:** Estimates of generalised additive mixed models predicting the probability of fox digging (*n* = 62,259 accelerometry bursts), while excluding female BJOV in 2022 for which degree of territoriality was uncertain. Model specifications are the same as those described in Table 2.

Component	Term	Estimate	SE	*z*	*p*
1. Parametric coefficients	(Intercept)	−0.58	0.45	−1.30	0.193
	Probability of encountering a neighbour	0.03	0.01	3.32	< 0.001
	Territorial yes/no [no]	−0.22	0.34	−0.63	0.530
	Sex [male]	−0.12	0.27	−0.43	0.666
	Prob encounter: Territorial yes/no [no]	−0.21	0.02	−9.32	< 0.001
**Component**	**Term**	**edf**	**Ref.df**	**Chi.sq**	***p***
2. Smooth terms	s(ToD)	7.51	8.00	9620.70	< 0.001
	s(day)	1.02	1.03	0.40	0.535
	s(year)	0.99	1.00	57,339.56	< 0.001
	s(day_factor, year)	34.34	45.00	381.55	< 0.001
	s(Fox ID)	17.76	18.00	4829.19	< 0.001

**TABLE A2:** Estimates of generalised additive mixed models predicting the probability of fox digging (*n* = 41,507 accelerometry bursts), while excluding locations falling within a 1 km inner buffer of study area boundaries. Model specifications are the same as those described in Table 2.

Component	Term	Estimate	SE	*z*	*p*
1. Parametric coefficients	(Intercept)	−0.56	0.41	−1.37	0.172
	Probability of encountering a neighbour	−0.03	0.01	−2.18	0.029
	Territorial yes/no [no]	−0.32	0.37	−0.88	0.379
	Sex [male]	0.00	0.29	0.01	0.992
	Prob encounter: Territorial yes/no [no]	−0.15	0.03	−5.20	< 0.001
**Component**	**Term**	**edf**	**Ref.df**	**Chi.sq**	***p***
2. Smooth terms	s(ToD)	7.30	8.00	6846.34	< 0.001
	s(day)	2.78	2.95	2.80	0.404
	s(year)	0.98	1.00	19,750.61	< 0.001
	s(day_factor, year)	32.71	45.00	258.49	< 0.001
	s(Fox ID)	17.69	18.00	2334.87	< 0.001

## Appendix C Inclusion of Nesting Goose Density in 2019

In 2019, fine‐scale nesting goose density was evaluated through a detailed field survey. As data were unavailable for 2022, we did not include nesting goose density in our model (Table 2). We verified whether including this covariate in the model affected results for 2019 data exclusively. Table A3 compares results from (A) a model including nesting goose density as a covariate and (B) a model without the covariate. The effects of probability of encounter, territoriality and their interaction are similar between models; thus, the omission of this covariate in our main model (Table 2) should not affect our main results and conclusions.

**TABLE A3:** Estimates of generalised additive mixed models predicting the probability of fox digging in 2019 (*n* = 17,736 accelerometry bursts) when (A) including nesting goose density as a covariate and (B) excluding it. Apart from effect of year, other model specifications are the same as those described in Table 2.

(A) Probability of digging in 2019, including covariate nesting goose density					
Component	Term	Estimate	SE	*z*	*p*
1. Parametric coefficients	(Intercept)	−0.87	0.30	−2.92	0.004
	Probability of encountering a neighbour	0.03	0.03	1.14	0.254
	Territorial yes/no [no]	−0.29	0.47	−0.63	0.529
	Goose nest density	0.26	0.02	13.21	< 0.001
	Sex [male]	−0.14	0.39	−0.36	0.719
	Prob encounter: Territorial yes/no [no]	−0.37	0.04	−8.30	< 0.001
**Component**	**Term**	**edf**	**Ref.df**	**Chi.sq**	***p***
2. Smooth terms	s(ToD)	6.02	8.00	663.87	< 0.001
	s(day)	3.27	3.68	10.28	0.025
	s(day_factor)	9.63	21.00	21.19	0.003
	s(Fox ID)	9.88	10.00	889.74	< 0.001

(B) Probability of digging in 2019, excluding covariate nesting goose density					
Component	Term	Estimate	SE	*z*	*p*
1. Parametric coefficients	(Intercept)	−0.82	0.29	−2.81	0.005
	Probability of encountering a neighbour	0.04	0.03	1.46	0.143
	Territorial yes/no [no]	−0.41	0.46	−0.91	0.363
	Sex [male]	−0.13	0.39	−0.34	0.732
	Prob encounter: Territorial yes/no [no]	−0.41	0.04	−9.30	< 0.001
**Component**	**Term**	**edf**	**Ref.df**	**Chi.sq**	***p***
2. Smooth terms	s(ToD)	6.07	8.00	711.07	< 0.001
	s(day)	2.75	3.11	6.23	0.109
	s(day_factor)	9.73	21.00	22.32	0.003
	s(Fox ID)	9.88	10.00	873.47	< 0.001
